# Discovery of Web-Building Spiders in Gua Kelam, Perlis State Park, Malaysia

**DOI:** 10.21315/tlsr2024.35.1.5

**Published:** 2024-03-30

**Authors:** Johan Ariff Mohtar, Khadijah Hanim Abdul Rahman, Saktheswaran Nyanasilan, Nurul Ain Harmiza Abdullah, Fadhilah Mohamad

**Affiliations:** 1 Faculty of Chemical Engineering and Technology, Universiti Malaysia Perlis, Kompleks Pusat Pengajian Jejawi 3, 02600 Arau, Perlis, Malaysia; 2 Centre of Excellence for Biomass Utilisation, Universiti Malaysia Perlis, Kompleks Pusat Pengajian Jejawi 3, 02600 Arau, Perlis, Malaysia; 3 Jabatan Perhutanan Negeri Perlis, Km.2, Jalan Kaki Bukit, 01000 Kangar, Perlis, Malaysia

**Keywords:** Cave, Gua Kelam, Spider, Cytochrome oxidase-I, Epigean, Gua, Gua Kelam, Labah-labah, Sitokrom oksida-I, Epigean

## Abstract

A cave represents a subterranean ecosystem that harbours a myriad of unique, peculiar, and secluded flora and fauna. These biotas have evolved with a wide range of ecological adaptations that allow them to thrive in harsh environments with limited light. Gua Kelam 1 constitutes part of the Gua Kelam limestone caves system in the Nakawan Range of Perlis State Park, Malaysia. Previous observations indicated that it harbours a plethora of spider species; however, their existence is still elusive as speleobiological studies remain unexplored. Herein, we identified the cavernicolous spiders found in the dark zone areas of Gua Kelam 1 through a complementary approach based on morphology and DNA barcoding. From the morphological analysis, we described three web-building spiders of JTKK2 and JTKK3 groups down to the species-level to belong to *Nephilengys malabarensis*, and *Orsinome vethi* except for *Pholcus* sp. from JTKK4 individuals. The molecular analysis of the cytochrome oxidase-I (COI) genes of JTKK2 and JTKK3 individuals showed that they exhibited a high degree similarity with *N. malabarensis* (98.3%), and *O. vethi* (100.0%), respectively except for JTKK4 individuals with only 91.4% homology with *P. kuhapimuk*. Phylogenetic analysis also generated a congruent tree, in which the identified species are well nested within the family Araneidae, Tetragnathidae, and Pholcidae. By this integral approach, the three spiders were determined as *N. malabarensis*, *O. vethi*, and *Pholcus* sp. These spiders are originally epigean in their habitat but uniquely thrive in Gua Kelam 1.

## Abstract (ms)

Gua mewakili ekosistem bawah tanah yang menyimpan pelbagai flora dan fauna yang unik, pelik dan terpencil. Biota ini telah berevolusi dengan pelbagai adaptasi ekologi yang membolehkan mereka berkembang biak dalam persekitaran yang sukar dengan cahaya yang terhad. Gua Kelam 1 merupakan sebahagian daripada sistem gua batu kapur Gua Kelam di Banjaran Nakawan Taman Negeri Perlis, Malaysia. Pemerhatian terdahulu menunjukkan bahawa ia mempunyai banyak spesies labah-labah; bagaimanapun, kewujudan mereka masih sukar dijumpai kerana kajian speleobiologi masih belum diterokai. Di sini, kami mengenal pasti labah-labah gua yang terdapat di kawasan zon gelap Gua Kelam 1 melalui pendekatan pelengkap berdasarkan morfologi dan pengekodan DNA. Daripada analisis morfologi, kami mengenal pasti tiga labah-labah pembina sawang dari kumpulan JTKK2 dan JTKK3 hingga ke peringkat spesies tergolong sebagai *Nephilengys malabarensis*, dan *Orsinome vethi* kecuali spesies *Pholcus* daripada individu JTKK4. Analisis molekul gen sitokrom oksida-I (COI) bagi individu JTKK2 dan JTKK3 menunjukkan mereka mempamerkan kemiripan jujukan yang tinggi dengan *N. malabarensis* (98.3%), dan *O. vethi* (100.0%), masing-masing kecuali individu JTKK4 dengan hanya 91.4% homologi dengan *P. kuhapimuk*. Analisis filogenetik juga menghasilkan pokok yang terakur, di mana spesies yang dikenal pasti digolongkan dengan betul dalam keluarga Araneidae, Tetragnathidae, dan Pholcidae. Dengan pendekatan bersepadu ini, tiga labah-labah tersebut ditentukan sebagai *N. malabarensis*, *O. vethi*, dan spesies *Pholcus*. Labah-labah ini pada asalnya bersifat epigean di habitatnya tetapi secara uniknya hidup di dalam Gua Kelam 1.

**Highlights**

- An integral approach based on morphology and DNA barcoding reveals three species of cavernicolous spiders from Gua Kelam 1.
- *Nephilengys malabarensis*, *Orsinome vethi*, and *Pholcus* sp. are web-building spiders.
- Commonly epigean in origin, the spiders uniquely adapt to the subterranean life in the dark zone of the cave illuminated with artificial dim lights.
- Despite thriving in the dark zone of Gua Kelam 1, these spiders do not exhibit any troglobiomorphic characteristics.

## INTRODUCTION

A cave is a hypogean chamber that is formed due to natural phenomena such as volcanic eruptions, glaciers, sand, waves and ground water activities. A majority of caves in the world, known as solution caves, are created in the landscape largely encompassed by carbonate rocks, e.g., limestone and dolomite (Das *et al*. 2007). The presence of slightly acidic ground water in proximity gradually dissolves the minerals as it percolates through the surface cracks. Over thousands of years, the corrosion eventually creates a network of underground passages; the larger ones that can fit humans are usually dubbed as caverns.

Caves are non-homogeneous in their ecology and can be divided into three biological zones: the entrance zone rich in sunlight with abundant epigean (above ground) animals; the twilight zone in very low levels of light with less animal species, and the dark zone with no reachable lights and the most nutrient-poor zone (Culver & Pipan 2009; Hesselberg *et al*. 2019). A cave has been regarded as an ideal “evolutionary laboratory” that supports troglofauna to adapt in darkness. Of these, troglobiomorphic spiders have become one of the successful cavernicoles; they thrive in different cave environments either as trogloxenes (sporadic hypogean), troglophiles (facultative hypogean), or troglobites (strictly hypogean bound) (Barr 1967; Cardoso *et al*. 2011). Troglobitic spiders have drawn the attention of many evolutionary scientists as they possess bizarre morphological body adaptations such as depigmentation and eye loss (Cardoso & Scharff 2009; Bloom *et al*. 2014; Rodrigues *et al*. 2020). Thus, they are perceived as highly potential models for probing into the evolution of life in extreme habitats. At present, it is estimated that there are at least 48 spider families consisting of troglobiomorphic species, e.g., the first eyeless huntsman spider (Sparassidae) from Laos was discovered in 2012 (Mammola & Isaia 2017). Of these, approximately 1,000 are classified as troglobionts (Ribera 2004).

Gua Kelam is a limestone cave system located in the continuous belt of the Nakawan Range that constitutes part of the Setul Formation (Jasin 2010). It is situated in Perlis State Park, at the edge of Kaki Bukit, Perlis, under the authority of the Perlis State Forestry Department. The park has been designated as a recreational destination for tourists (Mokhtar *et al*. 2012; Syamsul *et al*. 2012). Historically, the area was explored for tin ore mining in the 19th century. As a whole, the Gua Kelam cave system comprises several interconnected caves such as Gua Kelam 1, Gua Kelam 2, Gua Ikan, Gua Tikus, Gua Kambing, Gua Baba, Gua Lo Po Sang, Gua Foh Thye, and the recently discovered Gua Kelam 3 (Price 2011; Kamarudin *et al*. 1998). In 2020, Gua Kelam has been charted as a geosite by the Department of Mineral and Geoscience Malaysia.

**Figure 1:**
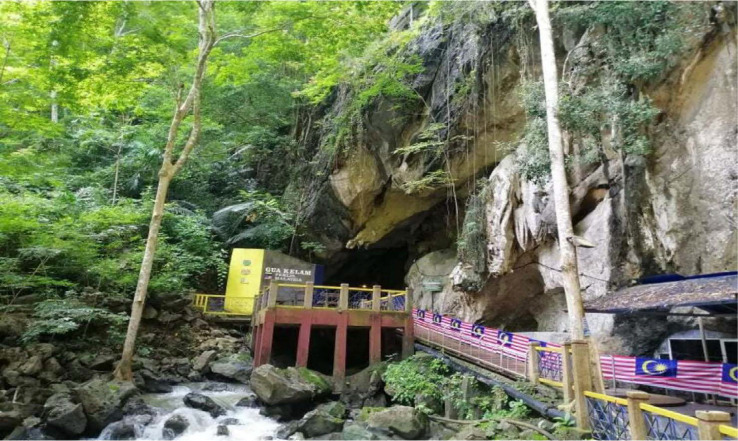
The entrance of Gua Kelam 1 with its flowing stream at the bottom.

Gua Kelam 1 is a through-cave with a stream under it, approximately 370 m in length (Fig. 1). The entrance lies at the base of the limestone range and exits towards a lake on the other side of the cave. A suspended bridge was formerly constructed along the cave as a means of access for the locals to travel and for tin miners to transport supplies and tin ore (Kamarudin *et al*. 1998). Nowadays, Gua Kelam 1 has gained immense popularity as a tourist destination fitted with artificial lightings that operate daily from 8:00 a.m. to 6:00 p.m. for attraction. Over the years, while the bridge has introduced pedestrian traffic, the cave system still supports several subterranean animals such as bats, cave toads, crustaceans and arthropods. However, little is known of its fauna until recently. Gua Kelam 1 has been reported to accommodate various species of spiders, but no study has been conducted to investigate its species biodiversity (Kamarudin *et al*. 1998; Price 2011). Therefore, the aim is to reveal the troglofauna of cave-dwelling spiders in Gua Kelam 1.

Morphology based identification of spiders can be sometimes difficult to achieve due to sexual dimorphism and variations in color and genitalia structure that often resulted in inaccurate identification (Jocqué 2002; Huber & Gonzalez 2001). With the advancement of DNA technology, DNA barcoding using standard genetic markers such as cytochrome oxidase-I (COI) has been integrated in species identification for genus-species clarification, particularly in taxa with complex morphological characters (Nicholls *et al*. 2010; Nicholls *et al*. 2012). In this study, an integral approach of morphological characterisation and DNA barcoding was implemented.

## MATERIALS AND METHODS

### Sampling of Spiders

**Figure 2:**
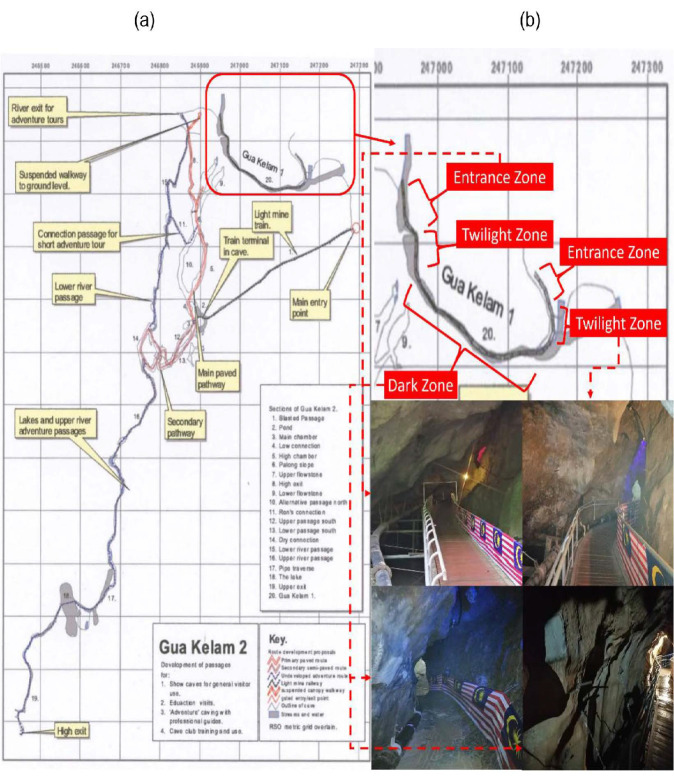
(a) Map of Gua Kelam cave system indicating the location of Gua Kelam 1; (b) close-up map of Gua Kelam 1 depicting the entrance, twilight, and dark zones with their respective *in situ* images. Scale not given. (Source: Malaysian Nature Society Cave Group (MNSCG) in association with Perlis Climbers 1996, Retrieved from Perlis Climbers, 2017)

Spider collection was performed twice during the rainy season on 17 September and 20 October 2020. We divided the cave cavity into three speleobiological zones according to the presence of sunlight (Fig. 2b) (Hesselberg *et al*. 2019). Sampling was focused on the dark zone area with the assistance from the park rangers. Despite being designated as the dark zone as it was devoid of sunlight, the area was eventually illuminated with artificial dim lights. With the use of a fabricated sweeping net, spiders were collected either from the cave wall, crevices, and along the bridge railings. In addition, the temperature and humidity were also recorded using a digital hygrometer. All spiders were kept in individual plastic containers prior to transferring to the Tissue Culture and Biomolecular Laboratory at UniCITI Alam Campus, Universiti Malaysia Perlis, Perlis. All specimens were deposited in the Collection Unit of Tissue Culture and Biomolecular Laboratory (TCBL).

### Morphological Characterisation

Initially, the spider specimens were separated into three groups that were designated as JTKK2, JTKK3 and JTKK4 based on morphological differences using photographic identification (Koh & Bay 2019). Next, one representative from each group was selected for a stereo-microscopic analysis (Olympus, Japan) and further characterised according to Kuntner (2007), Huber (2011), Alvarez-Padilla and Hormigo (2011) and Caleb *et al*. (2018). The measurements of total body length, prosoma, opisthosoma, and each leg segment were recorded. All measurements were in millimeters. Diagnostic photos were captured either with a S3CMOS microscope eyepiece camera mounted on an Olympus SZ51 stereo microscope (Olympus, Japan) using ToupView software (Ver. 3.7.0, ToupTek, Hanzhou, China), or a D7100 camera using a Micro-Nikkor lens 60 mm (Nikon, Japan).

### Genomic Extraction

Prior to genomic DNA (gDNA) extraction, the spiders were starved for 48 h and succumbed to deep-freezing for 5 min at −80°C. Following washing, gDNA was extracted from four legs of individuals of JTKK2 (*N* = 1) and JTKK3 (*N* = 1), respectively. For JTKK4 individuals, the whole body was used (*N* = 1) as it was soft-bodied and fragile. The extraction was performed using a gDNA extraction (tissue) kit (RBC, South Korea), according to the manufacturer’s instruction with slight modifications. Briefly, samples were homogenised in 200 μL of GT lysis buffer with the addition of 30 μL proteinase K prior to incubation at 60°C for 30 min, and further incubated with 200 μL QCB buffer for 20 min. Following a 2-min centrifugation at 13,000 rpm, the supernatant was recovered and pre-treated with RNase at 10 mg/mL for 10 min at room temperature prior to mobilising onto a GD column. After washing the matrix with 200 μL absolute ethanol, 400 μL W1 buffer, and 600 μL wash buffer, gDNA was eluted in 30 μL elution buffer and further treated with RNase. All gDNA were stored at −20°C until further use.

### Amplification of COI Gene

A set of primers were designed to amplify the mitochondrial cytochrome oxidase subunit 1 (COI) gene using a Primer-BLAST^®^ provided by the National Centre for Biotechnology Information (NCBI) (Rowan & Paul 2005). PCR amplification was performed on a standard benchtop thermocycler (Bio-Rad, USA) using a Master Mix reagent (Promega, USA). Briefly, 25 μL reaction mixture was prepared by mixing 1 μL gDNA template, 12.5 μL GoTaq^®^ Green Master Mix, 0.25 μL of each respective primer (10 μM), and 11 μL nuclease-free water. The cycling conditions were as follows: denaturation at 94°C for 60 s followed by 30 cycles of 94°C for 45 s, 50°C for 45 s, 72°C for 30 s, and ended with a final extension at 72°C for 5 min. Each DNA sample was tested with a universal forward primer, LCO1490A: 5′–GGTCAACAAATCATAAAGATATTGG–3′, and a specific reverse primer, chelicerate reverse 2 (CR2): 5′–GGATGGCCAAAAAATCAAAATAAATG–3′ in the following combination: LCO/CR2. The resulting amplicons were visualised on 1% agarose gel using GelDoc Imager (Biorad).

### Phylogenetic Analysis

PCR amplicons were purified using a MEGAquick-spin™ Plus Total Fragment DNA Purification Kit (iNtRON Biotechnology, South Korea). DNA sequencing was subjected to Sanger sequencing and the resulting sequences were analysed using bioinformatic tools. Following sequencing trimming using BioEdit (v. 7.2), the alignment was performed on nucleotide BLAST (BLASTn). The total of 35 related sequences were chosen for the construction of phylogenetic tree (see Table 1). The sequences were aligned using the MUSCLE method (Edgar 2004) on MEGA X (v. 11.0). General Time Reversible (GTR) model was used to calculate the pairwise genetic distances between spider species (Nei & Kumar 2000). The phylogenetic tree was estimated using Maximum Likelihood Statistical Method with 1,000 bootstrap replicates generated by MEGA X (v. 11.0) (Kumar *et al*. 2018).

## RESULTS AND DISCUSSION

**Figure 3:**
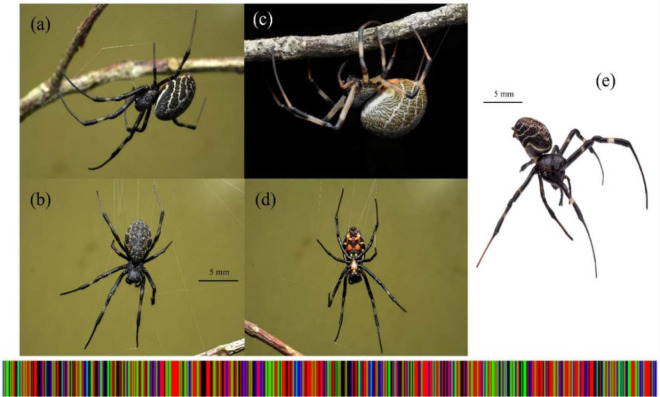
*Nephilengys malabarensis*. (a) lateral habitus; (b) dorsum habitus; (c) lateral habitus of typical brown morph; (d) ventral habitus; (e) frontal-lateral habitus. DNA barcode is presented below the illustrations.

**Figure 4:**
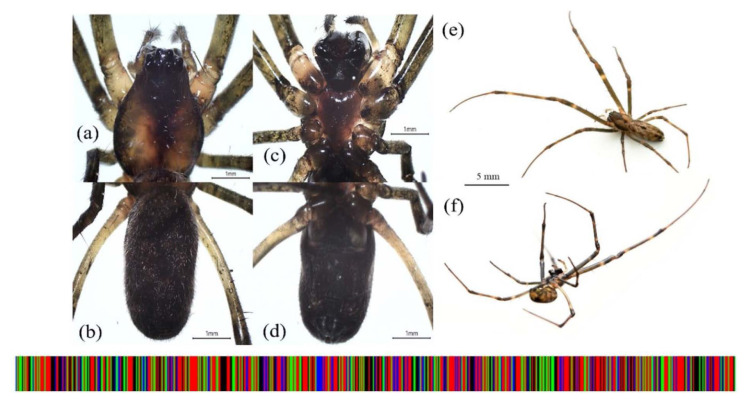
*Orsinome vethi*. (a) dorsal carapace; (b) dorsal opisthosoma; (c) ventral sternum; (d) ventral opisthosoma; (e) dorsal habitus; (f) lateral habitus. DNA barcode is shown below the illustrations.

**Figure 5:**
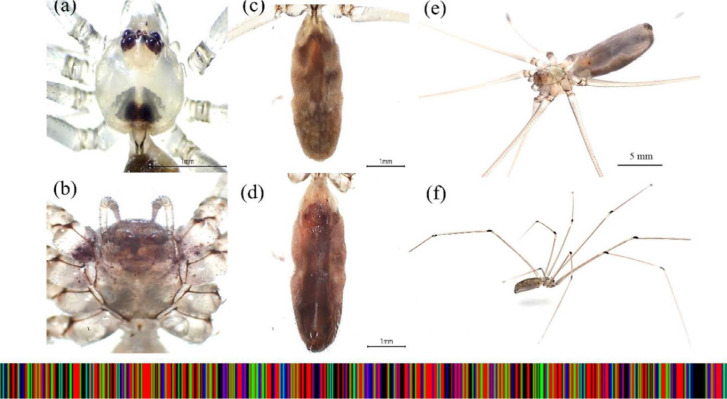
*Pholcus* sp. (a) dorsal carapace; (b) ventral sternum; (c) dorsal opisthosoma; (d) ventral opisthosoma; (e) ventral habitus; (f) lateral habitus. DNA barcode is shown below the illustrations.

During the sampling period, a total amount of 24 individual adult spiders were randomly collected from the dark zone region of Gua Kelam 1 and sorted into three morphologically similar groups, namely JTKK2, JTKK3 and JTKK4. Based on the photographic record and taxonomic characters, we identified the individuals from JTKK2 (*N* = 6), JTKK3 (*N* = 8) and JTKK4 (*N* = 10) groups to belong to *Nephilengys malabarensis* (Kuntner 2007) (Fig. 3), *Orsinome vethi* (Alvarez-Padilla & Hormigo 2011; Caleb *et al*. 2018) (Fig. 4) and *Pholcus* sp. (Huber 2011; Nentwig *et al*. 2023) (Fig. 5), respectively. These groups represent three species of web-building spiders of the family Araneidae, Tetragnathidae and Pholcidae.

### Morphological Description

#### *Nephilengys malabarensis* (JTKK2)

The studied sample of *N. malabarensis* (JTKK2) could be easily characterised by the below-mentioned characters: Total body length (mm) = 21.50; *Prosoma* (6.82 long and 5.01 wide); *Opisthosoma* (11.80 long and 7.15 wide). The whole body were uniformly black with black chelicerae. The carapace was dark with bright orange sternum in live animal. *Appendages*: The legs and palps were annulated white and black: coxae, trochanters, distal femora, patellae, distal tibiae, metatarsi, tarsi black, proximal femora, tibiae white. The length of Leg I segment was as followed: Total length (mm) 10.0 (femur 2.9, patella 0.8, tibia 2.2, metatarsus 2.9, tarsus 1.2). The dorsum was white with brown dots and the lateral opisthosoma contained dorso-ventral-longitudinal white-yellow bands. The venter was black with two large irregularly shaped pairs of orange patches. Another small pair of yellow dots were located on the venter between epigynum and pedicel. One pair of white dots were present around the spinnerets. Inside Gua Kelam 1, the samples of *N. malabarensis* were collected from the extensively overlapping vertical orb webs on the cave wall in the dark zone. The webs were built high towards the cave ceiling and the spider numbers were scarce. Some individuals were close to each other on the same web but territorial.

#### *Orsinome vethi* (JTKK3)

Meanwhile, individuals of *O. vethi* (JTKK3) were identified with the following characters: Total body length (mm) = 8.63; *Prosoma* (4.19 long and 1.85 wide); *Opisthosoma* (6.12 long and 4.07 wide). The carapace was slightly ochre with dark median stripe with radial lines and a pair of dark marginal bands while the sternum was ochre. The ocular area and the chelicerae were black. *Appendages*: The palps were light brown and darker at distal with little hairs. The length of Leg I segment was as followed: total length (mm) 15.1 (femur 4.6, patella 0.6, tibia 4.2, metatarsus 4.6, tarsus 1.1). The legs were greenish brown with little hairs and tiny spines. The femur and tibia were proximally and distally with yellow and dark bands. The opisthosoma was elongated and covered with numerous white spots. The pattern of white spots may vary among individuals. The abdomen turned dark when white spots contract in size if disturbed. The specimens were collected from extensive overlapping vertical and horizontal orb webs between the cave wall and bridge railings in the dark zone of Gua Kelam 1. The webs were only abundant in the area with flowing stream and contain individual spiders that tolerate close proximity with each other.

#### *Pholcus* species (JTKK4)

For *Pholcus* sp. (JTKK4), the spiders were identified with the following characters: Total body length (mm) = 4.62; *Prosoma* (3.55 long and 2.03 wide); *Opisthosoma* (1.28 long and 0.95 wide). The carapace was pale yellow with horseshoe-shaped grey-black posterior mark. The ocular area was black and elevated with each eye triad on low hump. The sternum and the chelicerae were pale yellow. *Appendages*: The palps were pale yellow with little hairs. The length of Leg I segment was as followed: Total length (mm) 10 (femur 2.9, patella 0.8, tibia 2.2, metatarsus 2.9, tarsus 1.2). The legs were covered with tiny spines and light brown. The femur was distally with black mark and patella and tibia-metatarsus joints were also black. The opisthosoma was elongated, and dark ochre and the dorsum was with heart-like shape in median stripe and posterior pale brown patches. The venter was with elongated transparent patch (pale brown). Inside Gua Kelam 1, the specimens were collected from irregular sheet webs built in cave crevices on the cave wall in the dark zone and the numbers of spider were scarce.

### Molecular Characterisation

**Figure 6:**
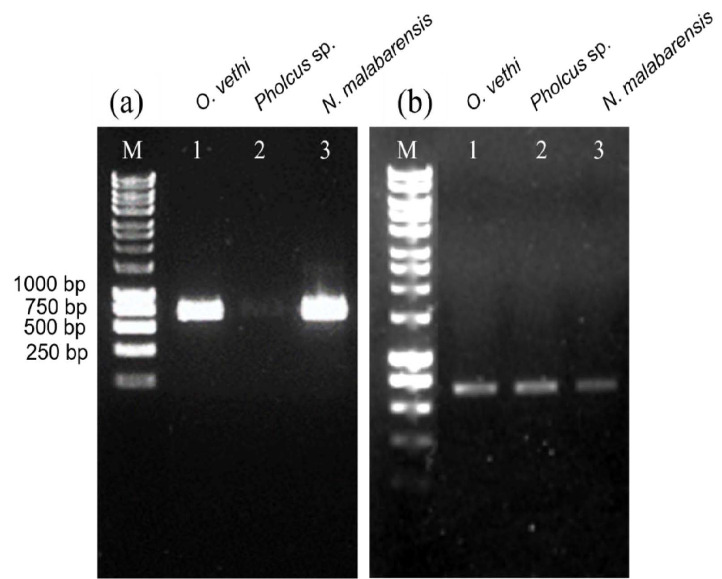
The integrity assay performed on 1% agarose gel. (a) ca. 700 bp COI amplicons amplified by LCO/CR2 with high intensity in all spiders except for *Pholcus* sp. (2); (b) reamplification of the COI gene for *Pholcus* sp. (2) by LCO/CR2 with comparable intensity to that of *O. vethi* (1) and *N. malabarensis* (3).

To further verify the species, the morphologically characterised spiders were investigated with molecular data using DNA barcoding approach. The combination of LCO/CR2 primer pair generated approximately 700 bp of amplification products for all three spider species as shown in Fig. 6a. It also showed that the intensity of the amplified products was high except for *Pholcus* sp. (2). Therefore, to resolve this, the amplicon was re-amplified to increase the product concentration by multiplying the cycle number, pooled, purified, and loaded on the agarose gel with the rest of the respective COI amplicons for integrity assessment (see Fig. 6b).

All amplicons from the three spiders were subjected to Sanger sequencing that resulted in the generation of good quality sequences of 674 bp, 675 bp and 628 bp for *N. malabarensis* (JTKK2), *O. vethi* (JTKK3) and *Pholcus* sp. (JTKK4), respectively. To infer the correct species, the refined amplicons were compared with homologous sequences in GenBank database. BLASTn search indicated that the COI genes of JTKK2 and JTKK3 displayed 98.3% (97% query coverage, *e*-value = 0.0) and 100.0% (88% query coverage, *e*-value = 0.0) homology to *N. malabarensis* (acc. no.: FJ607575) from Thailand and *O*. *vethi* (acc. no.: MK392942) from India, respectively, whereas, JTKK4 only shared 91.4% identity level (94% query coverage, *e*-value = 0.0) with *Pholcus kuhapimuk* (acc. no.: MG268830) from Thailand. All of the barcoded COI gene sequences in the current study were deposited in the GenBank under the accession number ON732862, ON732863, and ON732864 for *N. malabarensis* (JTKK2), *O. vethi* (JTKK3), and *Pholcus* sp. (JTKK4).

### Genetic Distance Analysis

**Figure 7:**
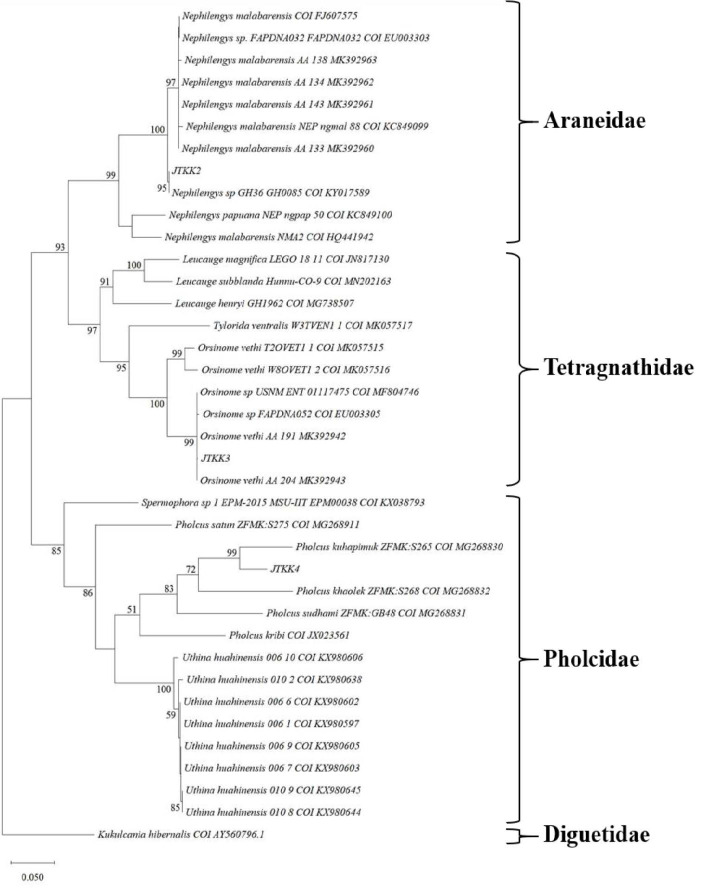
A maximum-likelihood phylogeny of COI genes from 35 published spiders including the three discovered species from this study, denoted as JTKK2 (*N. malabarensis*), JTKK3 (*O. vethi*) and JTKK4 (*Pholcus* sp.). The tree is rooted with *K. hibernalis* COI gene. Bootstrap values at nodes indicate the percent times recovered in 1,000 replicates and only values greater than 50% are shown. The scale bar depicts 0.05 substitution per site.

The phylogenetic analysis of the rooted maximum-likelihood tree (> 50% bootstrap) clearly recovered three clades for the spider species: Araneidae, Tetragnathidae and Pholcidae (see Fig. 7). Clustered within the family Araneidae, JTKK2 individuals formed a sister taxon with *N. malabarensis*, depicting a close relationship. JTKK3 individuals were also clustered in the same taxon with *O. vethi* in the corresponding clade, Tetragnathidae. Owing to similar morphological features and concordant genetic data to *N. malabarensis* and *O. vethi*, it was accurately confirmed that the individuals of JTKK2 and JTKK3 from Gua Kelam 1 were *N. malabarensis* and *O. vethi*, respectively. Although JTKK4 individuals were recovered in the clade of the family *Pholcidae*, its COI gene shared a low similarity percentage (91.4%) to *P. kuhapimuk*. Furthermore, given that the morphological features of many pholcids are barely distinguishable from each other (Bernhard *et al*. 2019), it was difficult to determine the species level of the spider. Thus, JTKK4 individuals in this study were treated as *Pholcus* sp. as they displayed elongated (cylindrical) opisthosoma, a common trait for genus *Pholcus* (Nentwig *et al*. 2023).

### Habitat of the Spiders

**Figure 8:**
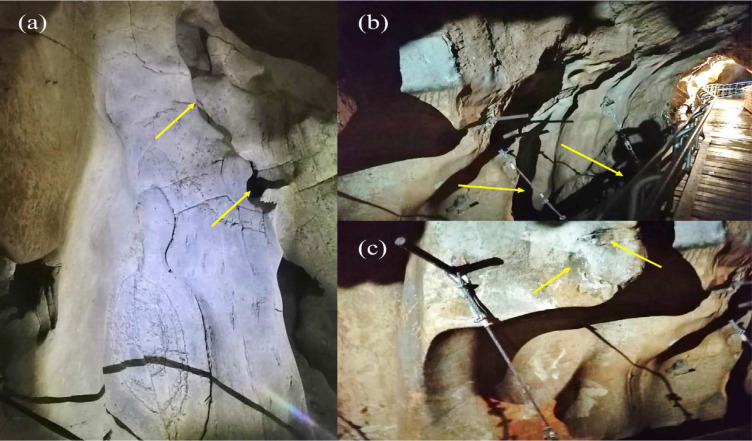
The natural surroundings of the spiders in the dark zone of Gua Kelam 1 as indicated by the yellow arrows. (a) The webbing structures on the elevated cave wall harbour a numbers of *N. malabarensis*; (b) *O. vethi* constructs extensive overlapping webs between the cave wall and bridge railings; (c) *Pholcus* sp. are scattered in the crevices or on the cave wall surface.

With the employment of the integral approach, the current work provided a morphogenetic identification of three web-building spiders, i.e., *N. malabarensis*, *O. vethi* and *Pholcus* sp. New DNA barcodes were generated for the three species from Gua Kelam 1 which have not been previously recorded. *N. malabarensis* is a Nephilid spider that builds large orb-webs with tubular retreat on dilapidated walls, tree trunks, or outcrops (Kuntner 2007; Dzulhelmi & Suriyanti 2015). It also thrives synanthropically around human residence (Koh & Bay 2019) and has a wide distribution in South, Southeast Asia: from India and Sri Lanka to the Philippines, Malaysia, Indonesia, Brunei, Singapore, North to China and North-East to Japan (Kuntner 2007). In the dark zone of Gua Kelam 1, the spiders constructed overlapping webs on elevated cave walls and some individuals could be found on the same large mesh where the numbers were more abundant in the twilight and entrance zones (Fig. 8a).

It is a newly discovered cave-dwelling colony of *N. malabarensis* in Malaysia. Previously, the cave population was reported at the entrance in Gua Niah, Sarawak, dubbed as *N. niahensis* (Deeleman-Reinhold 1989). However, Kuntner (2007) proposed that it was a junior synonym of *N. malabarensis*, as the diagnostic features were conspecific to the species in terms of the epigynum and size. Individuals of *N. malabarensis* from Gua Kelam 1 do not display troglobitic traits; instead, they exhibit melanism in high proportion compared to the typical brown morph (Fig. 3c). This finding was also supported by Kuntner (2007) who reported the existence of *N. malabarensis* dark morph from Java, Indonesia. Melanism is the darkening of body tissues due to an excessive melanin production that provides efficient regulation of thermal body temperature and protection from predators (Karpestam *et al*. 2012; Kuyucu *et al*. 2018). The phenotypic change related to darkening of species from cave upon exposure to dim light is a type of environmentally induced colour change that is less investigated (Oxford & Gillespie, 1998). Few studies have indicated that cave spiders inhabiting the twilight zone or in the artificial dimly lit cave region displayed body darkening (Legendre & Lopez 1973; Deeleman-Reinhold, quoted in Parker 1978). Since *N. malabarensis* individuals were scattered across the dark zone fitted with artificial lighting, this could possibly explain the existence of the melanic morph individuals. The physiological function of these colour variants is still unclear and it could be a form of adaptation. Nevertheless, such phenotypic colour change causes difficulty in identifying the species without the aid of DNA barcoding.

*O. vethi* is widely distributed from India, China, Vietnam, Laos, Malaysia to Indonesia (World Spider Catalog 2021). It is commonly found between rocks or vegetations over river in shady areas (Chrysanthus 1971; Koh & Bay 2019). They construct horizontal orb-webs containing 20 spirals, and 13 radii with open hubs. Strikingly, in the dark zone of Gua Kelam 1, they were observed to have built extensive overlapping vertical and horizontal webs between the cave wall and bridge railings over the flowing stream (Fig. 8b). In fact, the individuals are likely to display a colonial type of organisation trait as they can tolerate close vicinity to each other (Salomon *et al*. 2010). In Malaysia, no data on cave-dwelling *O. vethi* has been recorded, particularly in the dark zone; nonetheless, Eberhard (1992) reported that the genus thrives at the entrance and twilight zones in Tasmanian Caves, Australia. The species rarely penetrate the dark zone area and no troglobiomorphic traits were observed.

Pholcid spiders are common in well-covered microhabitats such as under rocks, tree holes and leaf litters (Koh & Bay 2019). They also thrive in caves, e.g., the genus *Uthina* are endemic in the twilight zones (Huber 2018; Huber *et al*. 2019) and display high endemism at the cave entrance (Yao *et al*. 2016). *Pholcus* sp. from Gua Kelam 1 was found to dwell in the dark zone. They constructed irregular sheet webs in the crevices or on the cave wall surface (Fig. 8c). The cave population, however, does not exhibit troglobiomorphic characteristics; thus, they seem not/slightly adapted to caves. This is corroborated by Huber (2018) that a large majority of cave-dwelling pholcids are not troglomorphic as they are only represented largely by two genera, *Anopsicus* and *Metagonia*.

This finding is unique as the species are generally associated with an epigean habitat. The presence of these web-building spiders in the dark zone is probably due to the spatial mobility from an external environment to the internal cave locality. Gua Kelam 1 is geographically a through cave with a stream running at the bottom. As juvenile spiders have the ballooning ability to use dragline silk that can catch air, and water-repellent legs to afloat on water, they can easily travel by means of wind and water currents (Hayashi *et al*. 2015; Lee *et al*. 2015) to finally settle down in the dark zone. The area stretches approximately 160 m from both sides of the cave and receives no sunlight except for artificial lighting on the cave walls. The lights are operational from 8:00 a.m. to 6:00 p.m. on a daily basis and these attract small aquatic flying insects such as beetles and mayflies to propagate in the water at the bottom. Kurniawan *et al*. (2018) showed that the presence of artificial lights in a cave’s dark zone increases the number of arthropods. As a result of food abundance, the spiders can possibly establish population for generations in the dark zone of Gua Kelam 1. In fact, the almost constant temperature along the area, between 27°C to 28°C and unusually high humidity of up to 99%, may also favour their reproductive cycle.

Moreover, Perlis receives heavy rainfall, particularly in November, during the northeast monsoon season from late September to December. This causes the river level at the bottom of Gua Kelam 1 to rise and flood the suspension bridge and large parts of the cave. The increasing water level may also contribute to the introduction of the spiders into the dark zone. For instance, a large number of aquatic crustacean troglobites have been discovered in regions previously flooded by the Late Mesozoic and Tertiary seas (Notenboom 1991).

We initially postulated that due to restricted dispersal and interchange among outside populations, these cave colonies may have become geographically isolated and undergone speciation. According to Mayr (1954), a population is exposed to the speciation process if it experiences a limited dispersal ability even if caused by small barriers such as rocks or seasonal flood; it becomes isolated. Ironically, the spiders do not exhibit troglobiomorphic changes despite their confinement in the cave. This raises a question to the extent of adaptation that the populations have undergone in the dark zone with artificial dim lightings. Future studies are needed to address the species speciation process in depth. Often, organisms which are more ecologically tolerant to different conditions can successfully colonise cave environments and are more likely to undergo troglobitisation. Nevertheless, one of the striking behavioural characteristics of these spiders except for *Pholcus* sp. observed was the construction of overlapping webs that contained solitary spiders in close proximity. The individuals of *N. malabarensis* and *O. vethi* were likely to display a degree of tolerance with each other on the overlapping mesh although they are generally solitary on single web in epigean environment (Kuntner 2007; Alvarez-Padilla & Hormigo 2011; Koh & Bay 2019). Whether or not this behaviour was a manifestation of a behavioural adaptation to the unusual habitat remains to be explored. We consider the three cave web-building spiders as facultative cavernicolous as they are able to adapt in the subterranean environment due to the availability of insects that are attracted to artificial lights fitted in the dark zone area of Gua Kelam 1. This discovery is significant for several reasons. First, no cave explored in the limestone region of Perlis has documented spider species biodiversity to date. Second, of all caves explored in the karst region in Malaysia for cave-dwelling spiders, only small fractions of troglophilic web-building species, including *Psechrus curvipalpus* (Pshceridae), *Psiluderces crinitus* (Ochyroceratidae), *Scytodes magnus* (Scytodidae), *Theridion rufipes* (Therididae), *Spermophora miser* (Pholcidae), and *Uloborus spelaeus* (Uloboridae), were previously described from Batu Caves, Selangor (McClure *et al*. 1967).

## CONCLUSION

In conclusion, a morpho-molecular identification of three spider species from Gua Kelam 1 is provided and their DNA barcodes are established in this study. The complementary approach based on morphology and DNA barcoding can be an effective tool in species identification of spiders. The discovery of the three cavernicolous web-building spiders, *N*. *malabarensis*, *O. vethi* and *Pholcus* sp. in a single cavern is astounding, as this sheds light on the spider biodiversity and ecosystem of Gua Kelam 1. Although the spiders are commonly epigean in origin, they have successfully established a subterranean colony inside the cave’s dark zone under favourable abiotic factors such as temperature and humidity. The cave populations are geographically isolated due to restricted dispersal. With air-catching dragline silk and water-repellent legs, these spiders may possibly be able to shift life from above ground to the cave environment by means of wind and water current, given by the look of the cave’s geography itself. Surprisingly, the described species do not display troglobiomorphic characteristics probably due to the effect of artificial dim lights. Future morphological and genetic comparison of epigean and hypogean populations of the related species, particularly the *Pholcus* sp. are highly needed as it will advance our understanding of their speciation in subterranean environments.

## Figures and Tables

**Table 1:** COI sequences of spiders with their corresponding accession numbers retrieved from GenBank database for phylogenetic tree construction.

No.	Species	GenBank accession no.
1	*Leucauge henryi* GH1962	MG738507
2	*Leucauge magnifica* LEGO 18 11	JN817130
3	*Leucauge subblanda* Hunnu-CO-9 COI	MN202163
4	*Nephilengys malabarensis* NMA2 COI	HQ441942
5	*Nephilengys malabarensis* AA 138	MK392963
6	*Nephilengys malabarensis* AA 134	MK392962
7	*Nephilengys malabarensis* AA 143	MK392961
8	*Nephilengys malabarensis* AA 133	MK392960
9	*Nephilengys malabarensis* COI	FJ607575
10	*Nephilengys malabarensis* NEP ngmal 88 COI	KC849099
11	*Nephilengys papuana* NEP ngpap 50 COI	KC849100
12	*Nephilengys* sp. FAPDNA032 COI	EU003303
13	*Nephilengys* sp GH36 GH0085 COI	KY017589
14	*Orsinome vethi* T2OVET1 1 COI	MK057515
15	*Orsinome vethi* W8OVET1 2 COI	MK057516
16	*Orsinome vethi* AA 191	MK392942
17	*Orsinome vethi* AA 204	MK392943
18	*Orsinome* sp USNM ENT 01117475 COI	MF804746
19	*Orsinome* sp FAPDNA052 COI	EU003305
20	*Pholcus khaolek* ZFMK:S268	MG268832
21	*Pholcus kribi* COI	JX023561
22	*Pholcus kuhapimuk* ZFMK:S265	MG268830
23	*Pholcus satun* ZFMK:S275 COI	MG268911
24	*Pholcus sudhami* ZFMK:GB48 COI	MG268831
25	*Spermophora* sp 1 EPM-2015 MSU-IIT EPM00038 COI	KX038793
26	*Kukulcania hibernalis* COI	AY560796.1
27	*Tylorida ventralis* W3TVEN1 1 COI	MK057517
28	*Uthina huahinensis* 006 9	KX980605
29	*Uthina huahinensis* 006 7	KX980603
30	*Uthina huahinensis* 010 9	KX980645
31	*Uthina huahinensis* 010 8	KX980644
32	*Uthina huahinensis* 006 6	KX980602
33	*Uthina huahinensis* 006 1	KX980597
34	*Uthina huahinensis* 006 10	KX980606
35	*Uthina huahinensis* 010 2	KX980638
